# Reproductive and Obstetric Outcomes after Fertility-Sparing Treatments for Cervical Cancer: Current Approach and Future Directions

**DOI:** 10.3390/jcm12072614

**Published:** 2023-03-30

**Authors:** Milan Terzic, Dinara Makhadiyeva, Jovan Bila, Mladen Andjic, Jelena Dotlic, Gulzhanat Aimagambetova, Antonio Sarria-Santamera, Antonio Simone Laganà, Vito Chiantera, Ivana Vukovic, Dusica Kocijancic Belovic, Slavica Aksam, Gauri Bapayeva, Sanja Terzic

**Affiliations:** 1 Department of Surgery, School of Medicine, Nazarbayev University, Zhanybek-Kerey Khans Street, 5/1, Astana 010000, Kazakhstan; 2 Clinical Academic Department of Women’s Health, National Research Center for Maternal and Child Health, Corporate Fund “University Medical Center”, Turan Ave. 32, Astana 010000, Kazakhstan; 3 Department of Obstetrics, Gynecology and Reproductive Sciences, University of Pittsburgh School of Medicine, 300 Halket Street, Pittsburgh, PA 15213, USA; 4 School of Medicine, Nazarbayev University, Zhanybek-Kerey Khans Street, 5/1, Astana 010000, Kazakhstan; 5 Clinic of Obstetrics and Gynecology, University Clinical Centre of Serbia, Dr Koste Todorovica 26, 11000 Belgrade, Serbia; 6 Faculty of Medicine, University of Belgrade, Dr Subotica Starijeg 8, 11000 Belgrade, Serbia; 7 Department of Biomedical Sciences, School of Medicine, Nazarbayev University, Zhanybek-Kerey Khans Street, 5/1, Astana 010000, Kazakhstan; 8 Unit of Gynecologic Oncology, ARNAS “Civico-Di Cristina-Benfratelli”, Department of Health Promotion, Mother and Child Care, Internal Medicine and Medical Specialties (PROMISE), University of Palermo, 90127 Palermo, Italy; 9 Department of Medicine, School of Medicine, Nazarbayev University, Zhanybek-Kerey Khans Street, 5/1, Astana 010000, Kazakhstan

**Keywords:** cervical cancer, infertility, fertility-sparing surgery, obstetrical complication, pregnancy outcome

## Abstract

Cervical cancer is one of the leading causes of cancer-related death in women of reproductive age. The established fertility-sparing approaches for the management of early-stage cervical cancer for women who plan pregnancy are associated with a decline in fecundity and an increased risk of pregnancy complications. This article aims to offer an overview of fertility-sparing approaches and the management of potential subfertility and pregnancy complications after these treatments. An extensive search for the available data about infertility and cervical cancer, fertility-sparing techniques in patients with cervical cancer, fertility treatment, obstetrical complications, and pregnancy outcomes in cervical cancer patients was completed. Fertility-preserving procedures such as loop electrosurgical excision procedure (LEEP), cold-knife conization, and trachelectomy in women diagnosed with cervical cancer can be considered as safe and effective treatments that preserve reproductive potential. Current fertility-preserving procedures, based on the balance of the oncological characteristics of patients as well as their desire for reproduction, allow one to obtain acceptable reproductive and obstetric outcomes in women treated for cervical cancer. Nevertheless, careful monitoring of pregnancies obtained after fertility-preserving procedures is recommended, since this cohort of patients should be considered at higher risk compared with a healthy population.

## 1. Introduction

Infertility is a global public health issue. Approximately 80 million people and about one-fifth of couples during their lifetime are affected by infertility worldwide [1,2,3]. In addition, infertility has a significant economic impact and is a multilayered stressor associated with anxiety, depression, and dysfunctions in marital and sexual relationships that affect quality of life [4,5,6].

Cancer treatment is one of the risk factors for premature menopause and female infertility [7]. Similar to other causes of infertility, cancer-related infertility has a negative influence on the psychological, social, and sexual functioning of patients [8,9,10]. However, today, cancer patients have the opportunity to access a variety of services for fertility preservation, and, in addition, fertility-supportive care is available to every patient at all stages of their cancer treatment [8].

Cervical cancer remains one of the leading causes of cancer-related mortality in young women aged 20–39 years despite the existence of human papillomavirus (HPV) screening and vaccination programs that can make it one of the most preventable cancers [11,12,13]. Cervical cancer has the fourth highest incidence and mortality in women worldwide [14]. Widely used treatments for cervical cancer include radical hysterectomy, with or without radiotherapy by external beam, vaginal brachytherapy, or chemo-radiotherapy of the pelvis [15]. Unfortunately, all these treatment options have the potential to lead to infertility [15]. Fertility-sparing surgery aims to preserve women’s fertility and may involve excisional cone biopsy and radical abdominal, laparoscopic, robotic, and vaginal trachelectomy [15] with or without pelvic and para-aortic lymph nodal staging. Such fertility-sparing approaches are only offered to women with cancer in the early stages, according to the Fédération Internationale de Gynécologie et d’Obstétrique (FIGO) staging system of cervical cancer, who plan pregnancy in the future [15]. Of note, fertility-sparing surgery offers similar overall and disease-free survival compared to radical hysterectomy [16]. At the same time, the fertility-sparing surgery applied for the management of cervical cancer is linked to an increased risk of pregnancy complications such as preterm deliveries, preterm premature rupture of membranes (PPROM), and low-birth-weight infants [15]. Because of the risks associated with preterm birth and PPROM, cervical cerclage is usually performed in patients after trachelectomy.

Cervical cancer can be diagnosed in a significant proportion of women before they have had the opportunity to bear children. Since the number of women with this type of cancer remains very high, and the prognosis for early-stage treatment is good, fertility preservation becomes of paramount importance when discussing management options with this cohort of younger patients, particularly bearing in mind that available standard cancer treatment leads to permanent infertility in almost all cases. This issue has been neglected for a long time, but fertility preservation is now considered an important quality-of-life issue for young patients with early-stage cervical cancer, and is now being studied in a more systematic and comprehensive manner. After undergoing targeted cancer treatment, women often regret not having received all the information they needed to make an informed decision about their fertility preservation options, which may then lead to depression, grief, stress, and sexual dysfunction.

This article aims to offer an overview of fertility-sparing approaches as well as the management of potential subfertility and pregnancy complications after these treatments in women affected by cervical cancer.

## 2. Materials and Methods

A search of the available literature on infertility and cervical cancer, fertility-sparing techniques in patients with cervical cancer, fertility treatment, obstetrical complications, and pregnancy outcomes in cervical cancer patients was performed. The search included Scopus, MEDLINE, Google Scholar, and PubMed databases, starting from 1990 to the beginning of 2023, using the following keywords, alone or in combinations, and MeSH IDs (whenever available): “infertility” (MeSH Unique ID: D007246; MeSH Unique ID: D007247), “infertility treatment”, “fertility preservation” (MeSH Unique ID: D059247), “fertility-sparing surgery”, “cancer”, “cervical cancer”, “uterine cervical neoplasms” (MeSH Unique ID: D002583), “cancer vaccines” (MeSH Unique ID: D019496), “Papillomavirus vaccines” (MeSH Unique ID: D053918), “infertility outcome”, “pregnancy outcome” (MeSH Unique ID: D011256), “obstetrical complication” (MeSH Unique ID: D007744), “prognosis” (MeSH Unique ID: D011379). Titles and abstracts of studies retrieved using the search strategy, as well as those from additional sources, were screened independently by three review authors to identify studies that could potentially meet the aims of this review. The full text of these potentially eligible articles was then retrieved and independently assessed for eligibility by a second review team consisting of three members. Any disagreement between the team members over the eligibility of particular articles was resolved through discussion with other collaborators. Peer-reviewed publications written in English and concerning infertility, cervical cancer, infertility, and pregnancy outcome in cervical cancer patients following fertility-sparing treatment were included in this review. Three authors independently extracted data from the articles about study features, inclusion criteria, types of intervention, and outcomes, using a pre-piloted standard form to ensure consistency. Any discrepancies which were identified were resolved through discussion with other team members. Due to the nature of the study findings, a narrative synthesis of the results from selected articles was opted in.

## 3. Results and Discussion

### 3.1. Fertility-Preserving Interventions in Cervical Cancer Patients

#### 3.1.1. Fertility-Sparing Surgery

**Figure 1:**
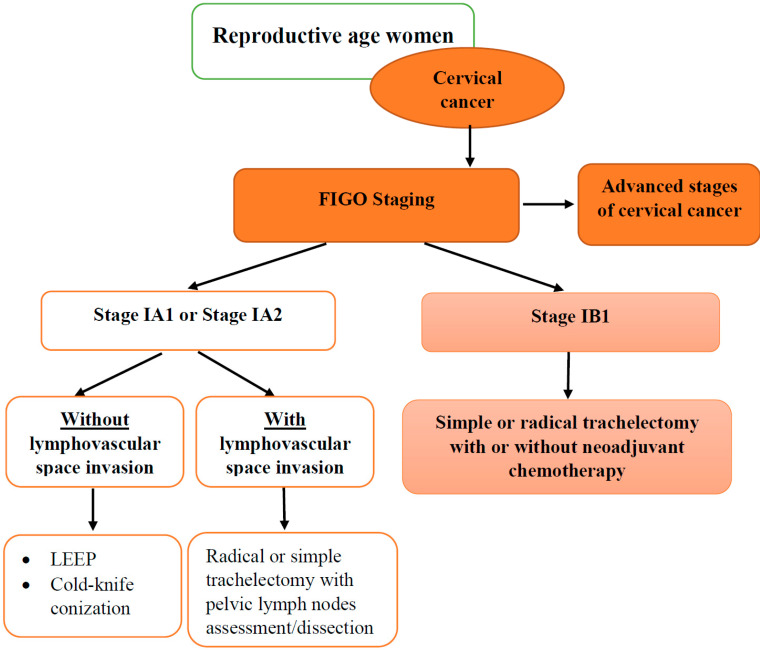
Fertility sparing surgical approach in early stages of cervical cancer. Figure legend: FIGO-Fédération Internationale de Gynécologie et d’Obstétrique; LEEP-loop electrosurgical excision procedure.

Early diagnosis of cervical cancer allows conservative surgical management, especially in young patients considering pregnancy in the future [16]. Considering that cervical cancer metastasizes to pelvic and para-aortic lymph nodes, their evaluation is imperative during fertility-sparing surgical procedures [16]. The loop electrosurgical excision procedure (LEEP) or cold-knife conization combined with endocervical curettage with negative surgical margins is a recommended fertility-sparing procedure for the management of stage IA1 of cervical cancer without lymphovascular space invasion (Figure 1) [17]. In the case of IA1 stage of cervical cancer with lymphovascular space invasion, a radical or simple trachelectomy with mapping of pelvic and sentinel lymph nodes is suggested [18]. In the case of IA2 stage without lymphovascular invasion, cold-knife conization is the method of choice. However, in the presence of lymphovascular invasion and exclusion of nodal involvement, radical or simple trachelectomy with pelvic lymph nodes dissection and sentinel nodes mapping is considered an option for cervical cancer [19]. Radical trachelectomy is recommended as a fertility surgical sparing method for patients with IB1 stage cervical cancer (Figure 1) [20]. Additionally, neoadjuvant chemotherapy followed by simple/radical trachelectomy or cold-knife conization is another potential fertility preservation strategy in cervical cancer management [21].

Overall, women diagnosed with squamous cervical cancer or adenocarcinoma who could become potential candidates for fertility preservation should meet the following requirements: (1) presence of an adequate ovarian reserve, (2) no evidence of lymph node metastasis, (3) cervical tumor size of <2 cm, and (4) stromal infiltration of <50% [22,23]. As cervical cancer is more frequent in women younger than 40 years old, the preservation of fertility is a significant public health issue for this group of women [22,23,24,25].

**Figure 2:**
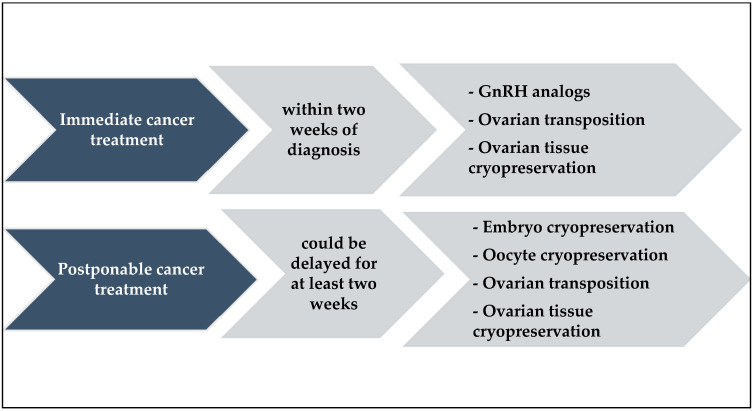
Non-surgical fertility-preserving approaches.

When fertility-sparing surgery is not an option for the treatment of cervical cancer, the following fertility-preserving approaches may be considered: ovarian suppression with the gonadotropin-releasing hormone (GnRH) analogs, ovarian transposition, oocyte cryopreservation, and ovarian tissue cryopreservation (Figure 2).

#### 3.1.2. Ovarian Suppression with GnRH Analogs

In women of reproductive age, advanced stages of cervical cancer necessitating neo-adjuvant and adjuvant chemotherapy and/or radiotherapy treatment, in addition to surgical management, would markedly reduce ovarian reserve and function. GnRH analogs inhibit the cellular turnover of ovarian cells, which are damaged during anti-cancer treatment, and therefore they are used for the prophylaxis of radiotherapy and/or chemotherapy-induced gonadotoxicity [26]. If there are potential contraindications for GnRH agonists, then an alternative option to impede accelerated follicle degradation secondary to chemotherapy or radiotherapy is to use GnRH antagonists [26]. A recent systematic review of GnRH analogue administration during chemotherapy with commonly used chemotherapeutic drugs for cervical cancer treatment (Paclitaxel and Cisplatin) showed a significant protective effect on ovarian reserve as reflected by patient’s post-therapy hormonal profile [27]. Although GnRH analogs are used in a fertility-sparing approach, they are applicable in urgent cases and when other non-surgical fertility-preserving options are not feasible [28,29,30]. Accumulating evidence suggests that factors influencing the efficacy of GnRH analogues as chemoprotective agents include the type and stage of cancer, the type of chemotherapeutics, pre-treatment of ovarian reserve, as well as a patient’s age [26]. However, further studies to evaluate the fertility outcomes of GnRH analogues and combinations of GnRH agonists and GnRH antagonists as gonadal-protecting agents are needed [26,31].

#### 3.1.3. Ovarian Transposition

Ovarian transposition as a fertility-preserving procedure involves the distancing of the ovaries in the abdomen away from the area of planned radiation therapy [31,32]. It is estimated that the success rate of ovarian transposition in terms of fertility preservation is approximately 90%. The transposition of ovaries makes transvaginal oocyte retrieval difficult during assisted reproduction treatment, and it is suggested that the transposition of one ovary should be combined with cryopreservation of the contralateral ovary [33]. Novel procedures of MRI-guided brachytherapy offer possibilities for selective radiation of the cervix without damage to the uterus and/or adnexa [34]. Bizzarri et al. (2022) reported that laparoscopic ovarian transposition prior to radiotherapy of cervical cancer can be a reproducible and standardized fertility-preserving procedure [35]. Taking into account that the metastasis of cervical cancer could be located in ovaries, there is a possibility of the fatal consequences of the transposition of ovaries affected by cervical cancer metastases. Fan et al. (2020) in their meta-analysis exploring risk factors for ovarian metastases in 18,389 cervical cancer patients with the stages IA to IIB (according to the FIGO classification) reported the incidence of ovarian metastases of up to 3.6%, depending on the type of cervical cancer [36]. Thus, adenocarcinoma of the cervix was associated with a three-fold higher risk of ovarian metastases compared to squamous cell carcinoma. According to the results of that meta-analysis, the risk factors for ovarian metastasis in cervical cancer patients are older age (more than 40 years old), bulky tumor (>4 cm), pelvic lymph node involvement, parametrial invasion, and invasion of corpus uteri [36]. Another study based on the analysis of 1160 women with transpositioned ovaries prior to cervical cancer treatment showed preserved ovarian function in nine (9) out of 10 patients undergoing surgery and brachytherapy and in six (6) out of 10 patients treated with external beam pelvic radiotherapy, with the development of ovarian metastases not exceeding 1% of all patients [37]. Thus, ovarian transposition may be considered as a valuable procedure in patients planning radiotherapy for cervical cancer management.

#### 3.1.4. Oocyte Cryopreservation

The American Society of Clinical Oncology (ASCO) and the European Society for Medical Oncology (ESMO) recommend oocyte and embryo cryopreservation prior to chemotherapy and/or radiotherapy for cancer patients planning pregnancy in the future [28].

Although still debated [16], oocyte cryopreservation is a well-established procedure that allows a predictable opportunity for future pregnancy based on the number of oocytes retrieved and stored [38]. Controlled ovarian stimulation (COS) with gonadotropin is required as an important step for this approach [16,38,39,40]. Currently, the probability of success in oocyte cryopreservation is related to the viability of oocytes after they are refrozen [16].

It is recommended that oocyte cryopreservation should be performed before anti-cancer treatment and patients should be fully informed about all risks and benefits of oocyte pick-up [39]. Campos et al. (2018) analyzed COS in 26 women with cancer, including two women with cervical cancer, and reported that ovarian stimulation with gonadotropins for urgent fertility preservation does not influence the number of metaphase II oocytes and therefore can be used as standard procedure for ovarian stimulation followed by oocyte pick up and cryopreservation [41]. Due to rapidly evolving techniques and protocols of ovarian stimulation during in vitro fertilization (IVF), the treatment allowed reproductive medicine specialists to utilize random-start ovarian stimulation that does not rely on a patient’s menstrual cycle [42]. This ensures no delay in initiation of a patient’s cancer treatment and maximizes reproductive chances of becoming pregnant upon successful treatment of the cancer [41].

Akel et al. (2020) compared the long-term outcomes of patients with gynecological cancers who underwent COS for fertility preservation [43]. Despite a delay in cancer treatment due to COS, it did not affect long-term outcomes in patients who underwent ovarian stimulation with gonadotropins compared to those without such approach [43].

However, multiple challenges prevent the suitability of COS and oocyte cryopreservation. The main problem with oocyte cryopreservation in patients with cancer is time constraints associated with the urgency for anti-cancer treatment that does not allow enough time for oocyte maturation during COS by gonadotropins [16,39,40]. In such cases, insufficient time for COS and the time required for oocyte maturation play a major role [16,40]. COS after hysterectomy due to cervical cancer is risky in terms of the ovarian response as ovarian blood supply is compromised after hysterectomy, thus responsiveness of the ovaries and the outcome of COS can be reduced [38].

Another challenge in oocyte cryopreservation for patients with cervical cancer is the possibility of triggering the spread of cancer during the COS and oocyte pick-up procedure [38,39]. However, some authors state that there is no scientific evidence behind this, thus, the risk is “worrying but theoretical” [38]. Moreover, cervical cancer is poorly responsive to sex steroid hormones; thus, it is unlikely to be harmful [38].

Finally, oocyte cryopreservation should be completed before chemo- and radiotherapy after informing patients about the risks of complications [16].

#### 3.1.5. Ovarian Cortex Cryopreservation

Ovarian tissue cryopreservation is a proposed fertility-preserving procedure for patients at risk of gonadotoxicity who require immediate anti-cancer therapy [40,44,45,46]. The advantages of ovarian cortex cryopreservation include tissue sampling independently of the menstrual cycle phases and the absence of hormone stimulation [40]. The method has been initially considered to hold an increased risk of unsuccessful subsequent ovarian tissue transplantation, as well as was believed to activate follicular development, thus compromising fertility success [47]. However, a recent systematic review showed promising results of ovarian tissue transplantation with regard to reproductive outcomes demonstrated by the restoration of ovarian function in up to 98% of the patients [48]. Pregnancy was achieved in over 80% of cancer-surviving patients undergoing ovarian tissue transplantation whereas the live birth rate reached 56% depending on the size of cryo-preserved ovarian tissue and the technique used for cryopreservation [48]. Another study showed a live birth rate following ovarian tissue cryopreservation and subsequent transplantation in 21% of cancer survivors with the application of IVF treatment and in 33% of cancer survivors with spontaneously achieved pregnancy [49]. Despite having a lower chance of achieving a live birth, the procedure is certainly worth considering for patients as it allows for spontaneous pregnancy [49]. However, the procedure still holds the risks of cancer recurrence with ovarian tissue transplantation. The evaluation of cervical cancer type and staging must be considered as metastases into ovarian tissue are common, especially in stage IIB (FIGO) of cervical adenocarcinoma [43,50], and it is mandatory to exclude ovarian metastases prior to considering ovarian tissue transplantation.

### 3.2. Fertility/Infertility after Cervical Cancer Treatment

Although the available fertility-sparing management procedures for patients with cervical cancer may achieve satisfactory results, there are potential complications related to these procedures such as cervical stenosis, vaginal bleeding from cervical vessels, and complications associated with the shortened cervix (spontaneous abortion, chorioamnionitis, PPROM, and preterm births) [51,52,53,54,55].

There is an active role of the cervix in supporting fertility/fecundity [38,56]. The fluctuations of the cervical mucus physiological characteristics through the menstrual cycle are influenced by sex steroids enabling the spermatozoa to overcome the functional cervical barrier only during ovulation. Spermatozoa may survive in the mucosal folds of the cervix and may later be “released” at the time of ovulation [38,56]. Considering those functional features are important for natural conception, cervical cancer treatment has an impact on natural fertility.

Multiple studies investigated the results of fertility-sparing treatment in the early stages of cervical cancer and considered the subsequent pregnancies and their outcomes [57,58,59,60,61,62,63,64,65,66,67,68,69,70,71,72,73,74,75,76,77,78,79,80,81,82,83,84,85,86,87]. Anderson et al. (2018) studied the pregnancy rate in women with a history of previous cancer and compared it to that of women from the general population (Table 1) [57].

The authors reported that the pregnancy rate in cancer survivors was lower compared to that of the general population, and cervical cancer survival was linked to the lowest chance of subsequent pregnancy [57]. Another study reported that approximately 55% of women with a medical history of stage I cervical cancer became pregnant and up to 70% of them gave a live birth [58]. After comparing different fertility-preserving strategies, the highest fertility and live birth rates were reported in the group of women treated with neo-adjuvant chemotherapy before the surgical procedure, in contrast to the women who underwent abdominal radical trachelectomy (Table 1) [58]. Nezhat et al. (2020) showed that radical vaginal and simple trachelectomy was associated with higher rates of conception [59].

One of the frequent complications of radical trachelectomy and cause of subsequent infertility is a cervical stenosis [60,61,62,63]. Even though there are approaches for the management of isthmic stenosis, the recanalization of cervical stenosis is often an unsuccessful procedure [63]. Nakajima et al. (2020) investigated the appropriate surgical procedures to prevent cervical stenosis after abdominal trachelectomy [64]. They observed that reconstructed uterine length between the uterine fundus and the vaginal end of neo-cervix shorter than 53 mm was associated with a higher chance of developing cervical stenosis.

The prevalence of infertility in patients after fertility-sparing management was reported in 33% to 57.7% of women who desired to conceive after abdominal radical trachelectomy [61]. Li et al. (2020) investigated reproductive and clinical outcomes after abdominal radical trachelectomy (Table 1) and reported that 58.6% of women did not plan to become pregnant immediately after abdominal radical trachelectomy [62]. In this study, in a group of women who tried to conceive, 17.4% achieved pregnancy [62]. The authors concluded that many women did not desire to conceive after abdominal radical trachelectomy, and the most common complications were cervical stenosis and fallopian tube obstruction, which resulted in a low pregnancy rate [62].

Severe cervical stenosis can be overcome using assisted reproductive technologies (ART) [65]. Hue et al. (2021) described the case of a 34-year-old woman who underwent robot-assisted radical trachelectomy and cerclage after the management of early-stage (IB2) adenosquamous carcinoma [66]. Approximately three months after surgery, she underwent ovarian stimulation but the transcervical embryo transfer was impossible due to the cervical stenosis. The authors performed transmyometrial embryo transfer under ultrasound monitoring, which resulted in a successful singleton pregnancy. In another study, authors reported a case of successful pregnancy and childbirth after direct intraperitoneal insemination in a 32-year-old nulligravida who previously underwent laparoscopic radical trachelectomy with paraaortic and pelvic lymphonodectomy due to stage IB1 cervical cancer [60].

The effects of chemo- and radiotherapy on ovarian function and the uterus should not be excluded. Paclitaxel, a worldwide-used microtubule stabilizing agent, has a gonadotoxic effect and leads to a reduction in anti-Müllerian hormone (AMH) levels [67]. There is also evidence of protective effects on ovarian function of GnRH analogs in combination with chemotherapeutics in the management of gynecological malignancies [68]. Although there are limited data on the impact of chemotherapeutics on the uterus, there is some evidence about the influence of chemotherapy on the uterine structure and function. It has been reported that chemotherapy is associated with a higher risk of preterm birth and a smaller uterus [69,70]. Cancer survivors treated with chemotherapy have lower pregnancy rates compared to the controls [71]. Pelvic radiotherapy as an adjuvant therapy impairs endometrium, myometrium, and uterine vasculature [70,72]. The impaired uterine volume and vasculature are associated with poor obstetric outcomes including a higher risk of preterm birth [70,73]. Accumulating evidence suggests that radiation doses to the uterus exceeding 25 Gy are associated with irreversible impairment of uterine structure and function, whereas doses of more than 45 Gy are linked with severe pregnancy complications or unsuccessful pregnancies [74].

Some studies show no influence on ovarian reserve and IVF outcomes in patients with cervical intraepithelial neoplasia [75,76]. However, Yang et al. (2022) conducted a retrospective cohort study including 111 infertile women, 37 with a history of early cervical lesions, and 74 controls to estimate the impact of early cervical lesions on IVF/ICSI cycle outcomes [77]. The authors reported a significantly higher rate of women with poor ovarian response in a group with early cervical lesions compared to controls [77]. The pregnancy rate and live birth rate were also lower in women with early cervical lesions than in age-matched women [77]. Tamauchi et al. (2021) reported that women who have undergone radical trachelectomy had lower estradiol concentration during the controlled ovarian stimulation cycle and a smaller number of retrieved oocytes compared to women with unexplained infertility or male factor infertility (Table 1) [78]. The authors concluded that radical trachelectomy was associated with a decreased ovarian response to controlled ovarian stimulation [78]. Another study by Nishio et al. (2013) estimated the reproductive outcomes in 114 women who had undergone radical abdominal trachelectomy. In this cohort of patients, 31 pregnancies were achieved in 25 patients (six patients had two pregnancies) [79]. Out of these 25 patients, 18 (72%) had infertility problems and required management with ART; 17 patients conceived with IVF and one patient with intrauterine insemination [79]. In the cited study, the pregnancy rate among patients who desired pregnancy was 36.2% (25/69) [79].

One important question that is posed following the success of cancer treatment and fertility-preserving procedures for cervical cancer remains the timing of fulfilling the reproductive goal of achieving a pregnancy and giving birth. It is recommended that a pregnancy should be planned six and nine months after loop LEEP and cervical conization, respectively, for women with cervical intraepithelial neoplasia [80]. Iwata et al. (2021) reported that one-, two-, and five-year cumulative pregnancy rates in women following fertility-sparing trachelectomy were 2.8%, 6.2%, and 17.4%, respectively [81]. There was a higher risk of cancer recurrence in women in the first 11 months after surgery. Thus, the attempt to conceive should be postponed up to 12 months after surgical treatment of early-stage cervical cancer [81]. The best predictors for calculating the chance of successful conception in cervical cancer survivors were determined to be younger age, presence of a partner, and the use of ART [81].

### 3.3. Pregnancy Course and Outcomes after Fertility-Sparing Management of Cervical Cancer

Despite an increase in the diagnosis of cervical cancer in women of reproductive age, a growing number of fertility-sparing procedures used in the management of early-stage cervical cancer, and known association of cervical cancer with increased risk of adverse fertilization and reproductive outcomes infertility, miscarriage, PPROM, and preterm birth, the evidence base guidance for fertility management of women surviving cervical cancer is lacking [82]. There are existing recommendations for the prevention of preterm birth for all patients after trachelectomy by administration of vaginal progesterone [82]. Cervical cerclage can be recommended to women with a history of preterm birth or late miscarriage, along with elective cesarean section as a choice for the mode of delivery [82].

Shinkai et al. (2020) investigated pregnancy outcomes and disease prognoses in women after vaginal radical trachelectomy who desired to preserve their reproductive ability [83]. They observed 28 pregnancies in 21 patients who underwent vaginal radical trachelectomy [83]. The median time from the treatment to pregnancy was 29.5 months after radical trachelectomy. Only one pregnant woman had cancer recurrence. The median gestational age at the time of delivery was 34 weeks. All deliveries occurred by caesarian section, while one in four of deliveries was an emergency procedure [83]. Kasuga et al. (2016) demonstrated a 44% pregnancy rate in women who tried to become pregnant after abdominal radical trachelectomy and the mean time to conception after abdominal radical trachelectomy was 3 years [88]. Two-thirds of pregnancies were achieved with ART treatment and resulted in a live birth [88].

The most common pregnancy complications after abdominal radical trachelectomy were extensive bleeding from the residues of the cervix and ascending infection [88]. Nishio et al. reported on 31 pregnancies in 25 women, with four women having had a miscarriage in the first trimester and one woman having had a miscarriage in the second trimester [79]. All women delivered by cesarean section, with 5 having had a delivery in the second trimester and 17 having had a delivery in the third trimester [79]. Only 23.5% of women from the study had delivery ≥37 weeks of gestation [79]. Ekdahl et al. (2021) analyzed long-term oncologic and reproductive outcomes after fertility-preserving robot-assisted radical trachelectomy [89]. The study included 166 women of which 149 have had robot-assisted radical trachelectomy; the recurrence of the disease was recorded in 6% of this population [89]. The researchers observed 81 pregnancies that resulted in 76 live births, of which 86% occurred after 32 weeks of gestation and 54% at term [89]. Tsaousidis et al. investigated reproductive outcomes in 23 women who had undergone large conization, a potentially safe and least invasive fertility-preserving procedure compared to radical trachelectomy [90]. All of the women attempting to conceive became pregnant, with most of them achieving pregnancy spontaneously. There was no relapse of cervical cancer during the follow-up period (2.6–8 years) [90].

It is crucial to achieve the right balance between oncological safety and cancer treatment outcome on one side and the reproductive ability and pregnancy outcome on the other [30,38]. Surgical treatment of cervical cancer can lead to cervical incompetence with the progression of pregnancy [91]. The short or absent cervix is linked to a higher chance of intrauterine infections and PPROM [92]. Kyrgiou et al. (2016) reported a higher relative risk of preterm birth in women who underwent large LEEP or cold-knife conization [93]. Additionally, the risk for preterm birth correlates with an increase in the depth, volume, and size of the cervical coning [94]. There are differences in the incidence of preterm birth after different fertility-sparing procedures, with the highest incidence observed in laparotomic radical trachelectomy, laparoscopy-assisted radical trachelectomy, and Dargent procedure, and the lowest incidence after simple trachelectomy or cone resection [58].

There are established procedures to prevent preterm delivery and to improve pregnancy outcomes in women after trachelectomy such as the use of antibiotic prophylaxis during pregnancy [95], cervical cerclage [96], routine cervicometry [97], strict rest with tocolytic therapy and vaginal irrigation as well as artificial maturation of the fetal lung by maternal corticosteroid administration [98].

Ekdahl et al. (2021) assessed possible factors associated with premature delivery in pregnancies achieved after robotic radical trachelectomy [99]. Factors such as previous vaginal deliveries, postoperative “non-pregnant cervical length”, uterine arteries preservation, and usage of oral metronidazole/no sexual intercourse regime during the second trimester were analyzed [99]. It has been reported that only oral metronidazole/no sexual intercourse regime had a four-fold reduction in second-trimester miscarriage and premature delivery [99].

Different techniques can be proposed as fertility-sparing surgery for women with cervical cancer, with various oncological and reproductive outcomes [100]. These outcomes have been extensively discussed in the recent systematic review by Morice et al. (2022), which included 5862 patients selected for fertility-sparing surgery [100]. The study assessed cervical cancer recurrence and pregnancy rates in women after fertility-preserving surgery [100]. The recurrence rates in women who were treated for stage IB1 cervical cancer were 4.1%, 4.7%, 2.4%, and 5.2% after simple conization/trachelectomy, radical trachelectomy by laparoscopic-vaginal approach, laparotomic or laparoscopic approaches, respectively [100].

The oncological outcomes in women with stage IB1 cervical cancer are quite similar across the different procedures [100]. The lowest pregnancy rate was observed in patients that underwent radical trachelectomy by laparotomy (36%) [100]. The authors suggested that the choice between fertility-sparing procedures intended for the management of cervical cancer should be individualized and based on oncological data of patients, with a balance between the best cure and the best fertility-sparing method and reproductive outcomes [100].

### 3.4. Prophylactic and Therapeutic HPV Vaccines for Prevention of Cervical Cancer Recurrence after Fertility-Sparing Surgery

#### 3.4.1. Prophylactic HPV Vaccines

The role of high-risk HPV types in the development and progression of cervical cancer is one of the most significant events in global healthcare [101,102,103,104,105,106]. It initiated the development of prophylactic HPV vaccines for the prevention of HPV-related conditions, including cervical cancer [103,104,105,106,107]. Nowadays, there are four HPV vaccines available for the prevention of HPV infection and HPV-related conditions, including bivalent (Cervarix and Cecolin), quadrivalent (Gardasil), and nonavalent (Gardasil-9) vaccines [105,108,109,110,111,112,113,114,115,116,117,118,119,120,121,122,123,124,125,126,127,128,129]. Moreover, a novel 14-valent recombinant vaccine is currently under development [108]. It is expected to target nine HPV types overlapping with those in Gardasil-9 as well as an additional five types (HPV-35, -39, -51, -56, and -59) [112].

All currently available prophylactic HPV vaccines are based on L1 protein virus-like particles (VLP) [103,105,130,131,132,133]. The mechanism of action of HPV vaccines is explained by the induction of neutralizing antibodies, which prevent the viral invasion of the basal membrane [105,107,113,114]. Overall, the HPV vaccines show sufficient immunogenicity, a high degree of safety/low level of side effects [105,114], and with no increased risk of serious adverse effects being observed [105,107,114,115]. Moreover, multiple studies have reported a significant decline in the prevalence of high-risk HPV types and genital warts caused by HPV [105,116,117,118,119]. Recent research results suggest that HPV-vaccinated adults might have a lower risk of developing HPV-related cancers than those who were not vaccinated [120,121,122].

As previously discussed, women can receive excisional surgery to treat precancerous cervical lesions or early stages of cervical cancer when fertility preservation is desired [114]. However, despite LEEP or laser conization performed for treatment the high-grade squamous intraepithelial lesion (HSIL), up to 8% of women after these procedures may experience a relapse of the disease [114,123]. It could happen due to the persistence of previously caught high-risk HPV infection or because of a new episode of HPV infection [124]. Currently, the available literature data suggest that the administration of the prophylactic HPV vaccine before or after surgical management of premalignant cervical lesions might reduce the risk of recurrence [114,125,126,127]. Recent meta-analysis results suggest that adjuvant HPV immunization with prophylactic vaccines after surgical excision for CIN 2 or greater is associated with a reduced risk of recurrent cervical dysplasia caused by HPV-16 and HPV-18 types [127]. Thus, preventative HPV vaccination as adjunct management before or after surgical treatment can be utilized as an additional simple and effective method to prevent relapse of cervical lesions or cervical cancer in HPV-positive women treated with LEEP or conization [114,125,126,127,128].

Furthermore, the HPV FASTER protocol, which suggests complementary utilization of cervical cancer screening and vaccination in women older than the HPV vaccination target group, is fully in line with the idea of preventing HPV infection after the fertility-sparing approach [129]. This guideline offers HPV vaccination to females aged 9–45 years, or even 50 years, irrespective of their HPV infection status; thus, the vaccination can be implemented in women after fertility-sparing treatment for cervical premalignant and malignant lesions.

#### 3.4.2. Therapeutic HPV Vaccines

Despite the development and implementation of prophylactic HPV vaccines [105,106,134,135], there is still a considerable population suffering from high-risk HPV-related conditions, including premalignant cervical lesions and cervical cancer [134,135,136], especially in low- and middle-income countries (LMICs) due to multiple factors: (1) limited global vaccine availability [134]; (2) HPV vaccines’ limited cross-protection [105,130,131,132]; (3) no therapeutic benefit from prophylactic HPV vaccines [105,134,135]; (4) high cost due to cold chain maintenance requirements [105,130,132]; (5) delayed impact on cervical cancer incidence, which may only become apparent 15–20 years after the implementation of mass HPV vaccination [135,136].

Moreover, even if fertility-sparing treatment is successful and no relapse is observed, in many cases after LEEP or conization women may still suffer from postsurgical hemorrhage and adverse obstetric outcomes due to cervical trauma and decreased length (preterm birth and second-trimester miscarriage) [135]. In some cases, incomplete excision can occur with the HPV-affected pathologic cells remaining on site and leading to the recurrence of CIN [136]. Hence, there is a need for a medication/approach that can fully eliminate malignant cells. Moreover, for individuals diagnosed with persistent or recurrent cervical cancer, five-year survival is <20%, and there are few treatment options [104]. Therefore, there is a clear need for improved cervical cancer management by including a novel adjuvant treatment approach [104]. It drives an emergent need to develop therapeutic HPV vaccines that will target high-risk types with an immediate impact on women who are already infected, by preventing the progression of the premalignant cervical lesions as a potentially effective option [114,135,136].

The development of therapeutic vaccines represents a promising and interesting approach to the treatment of HPV infection and related cervical lesions [125,136]. During the recent decade, considerable efforts have been invested in the research and development of therapeutic HPV vaccines [114]. Some of these vaccines are already being tested in clinical trial phases 2 and 3 [134]. However, none of these vaccines are available for clinical practice and wide-spread implementation [114,135,136,137].

The mechanism of action of therapeutic HPV vaccines is based on the generation of cell-mediated immunity [133,137]. There are many types of therapeutic HPV vaccines under development which are classified based on vaccine platform technologies: bacterial vector and viral vector-based, DNA- and RNA-based, peptide- and protein-based, and dendritic cell-based vaccines [133]. Ongoing clinical trials investigating the efficacy of therapeutic HPV vaccines report spontaneous regression of CIN2 and CIN3 lesions occurring in more than 30% of cases [114,134]. This indicates that these vaccines, when introduced into clinical practice, may potentially be used for fertility-sparing treatment in early-stage cervical cancer patients. Vaccines (prophylactic or therapeutic) being applied as an adjunct treatment to fertility-sparing management could theoretically prevent new or recurrent infections and cervical disease relapse [114,125].

### 3.5. Study Strengths and Limitations

The main strength of our research is based on the comprehensive compilation of more recent and up-to-date information related to the fertility-preserving options for women with early-stage cervical cancer. Nevertheless, some limitations should be taken into account when interpreting the data: first, only papers written in the English language were included. Second, only a narrative synthesis was possible due to the heterogeneity of different techniques used for fertility preservation (surgery ± chemotherapy ± radiotherapy), different timing of the techniques, potential use of different methods of ART (COS protocol, ovulation induction, luteal phase support) vs spontaneous pregnancy, different outcomes analyzed. Third, most of the available studies included a limited number of women with the desire for pregnancy after fertility-sparing treatment for cervical cancer, as well as heterogeneity regarding the types of patients included (i.e., different staging and grading). Fourth, the maternal–fetal outcomes may have been significantly influenced by multiple variables, such as residual cervical length after conization/trachelectomy, potential use of cerclage (which can be performed by different techniques, such as McDonald or Shirodkar), potential use of prophylactic antibiotics, and different types of pregnancy monitoring. Finally, the available evidence does not allow one to draw a firm conclusion about the best timing and mode of delivery in case of different types of fertility-sparing treatment for cervical cancer.

## 4. Conclusions

The advances in early diagnosis and management of cervical cancer may lead to significant improvements in the expectancy and quality of life of women diagnosed with cervical cancer. However, the treatment of cervical cancer may impair female reproductive function and affect fertility in young women who plan pregnancy after cancer treatment. There is evidence that cervical cancer may affect ovarian reserve and response to controlled ovarian stimulation during ART. Based on clinical experiences with other cancers, personalized female fertility-preserving approaches such as fertility-sparing surgery and cryopreservation of oocytes and ovarian cortex may preserve reproductive ability and improve subsequent pregnancy outcomes in cervical cancer survivors.

The risk of cancer recurrence should not be neglected in women treated with fertility-sparing procedures, and they are generally advised to plan pregnancy six months up to two years after cancer treatment. Pregnancy achieved in cancer survivors must be vigilantly monitored and conservatively managed to reduce potential pregnancy and birth complications such as miscarriage, preterm delivery, and stillbirth. There remains a need for well-designed, prospective studies including a large number of patients with a longer period of follow-up in order to truly evaluate the impact of pregnancy on cervical cancer patients’ survival as well as the impact of different fertility-preserving procedures on pregnancy outcomes. In particular, this review highlighted that most of the available evidence on oncological, reproductive, and obstetric outcomes are based on the results of patients with stage IB1 (FIGO) cancer. Thus, further investigations on fertility-preserving management outcomes among women with stage IB2 and IIA1 cancer are required. These studies should consider oocyte or ovarian tissue preservation prior to starting neo-adjuvant chemotherapy with the aim to down-stage cervical cancer spread before conization/trachelectomy.

## Figures and Tables

**Table 1:** Fertility outcomes in patients after cervical cancer treatment with fertility-sparing approach.

Author	Year	Study Design	Cohort Size, Patients (n)	Patients’ Age (Years)	Cervical Cancer FIGO Stage	Procedure	Main Findings	Outcomes
Anderson et al. [57]	2018	Retrospective	23,201	≤39	early stages of cervical cancer	“Details of treatments received were not available”	Cancer was associated with a lower chance of pregnancy, adjusted HR 0.57 (95% CI: 0.53, 0.61) for women >5 years after cancer diagnosis.	The proportion of first singleton pregnancies after cancer that ended in a live birth was higher compared with the general population.
Shah et al. [61]	2018	Cross-sectional	39	25–37	IA1–IB1	Vaginal radical trachelectomy	Significant proportion of women with early-stage CC do not receive adequate reproductive counseling before ART, and many women undergoing ART experience complications that can negatively impact their fertility.	- The average time to pregnancy was 13 months; - The clinical pregnancy rate was 54% (20 pregnancies in 13 patients): 8 pregnancies were spontaneous and 12 required fertility assistance; 5 pregnancies resulted in first-trimester miscarriage.
Li et al. [62]	2020	Retrospective	360	16–53	IA1–IB1	Surgery + adjuvant therapy	Cervical stenosis or fallopian tube obstruction led to a low pregnancy rate after ART following the fertility-sparing treatment for CC.	- A total of 149 patients attempted conception after abdominal radical trachelectomy; - Infertility was reported in 57.7% cases (86/149), and half of the patients used ART; pregnancy rate in the cohort was 17.4% (26/149); - More than half of pregnancies reached the third trimester (three cases of first-trimester and sixth-trimester miscarriages were reported).
Shinkai et al. [83]	2020	Retrospective	71	23–46	IA2–IB1	Vaginal radical trachelectomy + pelvic lymphadenectomy	Both the obstetrical prognosis and oncological prognosis after vaginal RT have become favorable for pregnant patients after vaginal RT.	- A total of 28 pregnancies in 21 patients: 13 patients had spontaneous pregnancies, 7—with artificial insemination by husband or ART; - CS performed for all of them
Tesfai et al. [84]	2020	Retrospective	19	19–36	IB–IIA	Neoadjuvant chemotherapy + fertility-sparing surgery (trachelectomy)	Neoadjuvant chemotherapy with fertility-sparing surgery is a feasible and safe option in select patients CC IB–IIA. Unfavorable prognostic factors: - non-responsiveness, - non-squamous pathology.	- Out of 15 patients with a successful abdominal radical trachelectomy, 3 patients reported spontaneous pregnancies; - All patients with spontaneous pregnancies delivered at full term via the CS; - One patient required ART but developed a local CC recurrence and was treated with a hysterectomy.
Tamauchi et al. [78]	2021	Case-control	14 patients and 30 controls	29–40	early stages of cervical cancer	Vaginal radical trachelectomy	The response to controlled ovarian stimulation worsens after radical trachelectomy.	Cancer survivors after radical trachelectomy had lower mean estradiol levels during controlled ovarian stimulation and a smaller number of retrieved oocytes, and a higher dosage of gonadotropins compared to the control group.
Rendón et al. [85]	2021	Retrospective	23	20–37	cervical cancer of ≥2 cm to ≤6 cm (IB1–IIA2)	Neo-adjuvant chemotherapy + fertility-sparing surgery (abdominal and vaginal radical trachelectomy)	Neo-adjuvant chemotherapy followed by abdominal or vaginal radical trachelectomy in early-stage CC is a good option for fertility sparing in well-selected patients with cervical tumors ≥2 cm.	- A total of 10/23 (43.5%) became pregnant; - A total of 7 patients delivered 11 - Babies overall (three patients delivered twice); - There were 4 term deliveries and 7 preterm births; - Only 1 patient had a first-trimester missed abortion at 10 week-gestation.
Fanfani et al. [86]	2021	Retrospective	42	19–44	IA2–IB1	Cervical conization and pelvic lymphadenectomy	Cervical conization is feasible for the conservative management of women with stage IB1 cervical cancer desiring fertility.	- A total of 22 (52%) patients tried to conceive; - A total of 18 pregnancies occurred in 17 patients; - A total of 12 live births were reported (6 pre-term and 6 term pregnancies); - A total of 2 pregnancy losses were reported (1 first-trimester and 1 second-trimester).
Yamamoto et al. [87]	2022	Retrospective	42	28–36	IA1–IB1	Cervical conization followed by pelvic lymphadenectomy	Cervical conization combined with pelvic lymphadenectomy represents a feasible conservative management for well-selected patients with early-stage cervical cancer.	- A total of 66.7% (2/3) of those who tried to conceive became pregnant; - Overall, 6 pregnancies were achieved by 2 patients: 4 term pregnancies (all 4 delivered vaginally; 1 miscarriage; 1 patient was still on pregnancy follow-up at the time of the paper’s publication).

Table footnotes: CS—cesarean section; ART—assisted reproductive technology; CC—cervical cancer.

## Data Availability

Not applicable.
